# Chronic disease comorbidity and associated factors among cancer patients in eastern Ethiopia

**DOI:** 10.3389/fonc.2024.1368611

**Published:** 2024-04-23

**Authors:** Abdi Birhanu, Michael Shawel Lemma, Biruk Habtamu, Nahom Wondwossen Worku, Monas Kitessa, Shambel Nigusie, Galana Mamo Ayana, Yehenaw Tenaw, Selamawit Sete, Bedasa Taye Merga, Ibsa Mussa

**Affiliations:** ^1^ School of Medicine, College of Health and Medical Sciences, Haramaya University, Harar, Ethiopia; ^2^ Hiwot Fana Cancer Center, Hiwot Fana Comprehensive Specialized Hospital, Haramaya University, Harar, Ethiopia; ^3^ School of Pharmacy, College of Health and Medical Sciences, Haramaya University, Harar, Ethiopia; ^4^ School of Public Health, College of Health and Medical Sciences, Haramaya University, Harar, Ethiopia

**Keywords:** cancer, anemia, diabetes mellitus, hypertension, cardiovascular diseases, human immune virus, mental illness, eastern Ethiopia

## Abstract

**Background:**

The occurrence of long-lasting comorbidities makes cancer management and treatment challenging because of their overlapping poor prognosis. However, there are no data that show the burden of these chronic cases in patients with cancer in Ethiopia. Therefore, this study aimed to assess the burden of and the factors associated with chronic disease comorbidity among cancer patients in the eastern part of Ethiopia.

**Material and methods:**

A cross-sectional study was conducted on 422 patients with cancer admitted to the only cancer treatment center in eastern Ethiopia. A simple random sampling technique was employed to select the study participants. Data were extracted from the patients’ medical records using a checklist. The collected data were entered into the Epi-Data statistical software version 3.1 and then exported to STATA version 17 for analysis. Bivariate and multivariate logistic regressions were used to assess the association between the outcomes and the independent variables. Finally, adjusted odds ratios (AORs) with 95% confidence intervals (CIs) were reported. The statistical significance of the factors was indicated at a *p*-value <0.05.

**Results:**

Of the 422 eligible medical records identified, 419 (99.3%) were included for analysis. A total of 230 (54.8%, 95%CI = 50.0%–59.6%) patients with cancer presented with one or more chronic diseases. Of these comorbidities, anemia, hypertension, and cardiovascular disease were the most common diseases reported. Obesity at admission (AOR = 1.91, 95%CI = 1.10–3.61) had a significant association with the occurrence of comorbidities among patients with cancer.

**Conclusion and recommendation:**

The overall prevalence of comorbidity among patients with cancer was relatively lower than that reported in previous studies. Being obese was significantly associated with the occurrence of comorbidities. Attention should be given to the burden of chronic comorbidities among patients with cancer through researching, formulating policies, and improving community literacy to manage comorbidities. Thus, interventions for weight reduction and the early detection and treatment of the comorbidities could limit further complications and lower the incidence of other comorbidities.

## Introduction

Cancer is a significant public health concern worldwide, which has caused approximately 9.6 million deaths globally (1). Cancer is projected to result in 1.1 million new cases and cause 711,429 deaths in Africa in 2020 (2). Ethiopia is experiencing an increase in cancer cases, similar to other African nations. It is estimated that there are over 60,960 new cancer cases each year and over 44,000 deaths from the disease. Breast cancer has contributed 30.2% of all cancer cases in Ethiopia, while cervical cancer contributed 13.4% of all cancers. Another type of cancer is colorectal cancer, with 5.7% of the total types of cancer (3, 4). Most patients with cancer suffer from a long-lasting disease, usually called comorbidity (1, 5). A significant clinical problem for cancer management and treatment is the presence of persistent comorbid illnesses in various patients with cancer. When evaluating variations in the diagnosis and treatment of cancer, it is crucial to determine the nature of the existing comorbidities. Comorbidities could affect how healthcare is used, how cancer is diagnosed, and how treatment decisions are made (6, 7). Due to polypharmacy and diminished compensatory mechanisms, it can be difficult to treat patients with severe comorbidities, particularly older patients who are also experiencing typical physiological changes associated with aging. Comorbidities reduce the quality of life of patients with cancer and decrease the clinical outcomes (8). Previous studies suggested that the presence of comorbid illnesses is associated with age, gender, smoking status, ethnicity, inadequate levels of physical activity, and socioeconomic level (1, 9–15). A study showed that, in the USA, the prevalent comorbidities included cardiovascular disease (CVD), obesity, and metabolic disease; mental health concerns; and musculoskeletal disorders (16). From the total cancers, lung cancer (58%), kidney cancer (54%), stomach cancer (53%), and prostate cancer (51%) had a high estimated comorbidity prevalence (16–20). The results also showed that the mortality rates among cancer patients with comorbidities are higher than those without comorbidities (21). The limitation of earlier studies was the estimation of the prevalence of comorbidities in specific cancer types (1, 22, 23) rather than the overall comorbidity burden among cancer cases. Similarly, in eastern Ethiopia, there is little information on the prevalence of comorbidities. Therefore, this study aimed to assess the prevalence of comorbidities and their associated factors in patients with cancer who visited oncology centers in eastern Ethiopia.

## Materials and methods

### Study area, setting, and study period

A cross-sectional study was conducted at the Hiwot Fana Comprehensive Specialized Hospital (HFCSH) Cancer Treatment Center from March 1 to 25, 2023. The Cancer Treatment Center of HFCSH, which is in Harar, is the only specialized clinic where all newly diagnosed cancers are referred for further management in the eastern part of Ethiopia. The Hiwot Fana Hospital Cancer Treatment Center provides oncology services to all populations within the eastern part of Ethiopia.

### Population

All patients with cancer who were admitted to the HFCSH Cancer Treatment Center were considered a target population. Those admitted to the Cancer Treatment Center at HFCSH from October 1, 2021, to February 10, 2023, were included in the study. For this study, data on admission were used to characterize the patients’ profiles. All patients with cancer admitted to the Cancer Treatment Center since it started providing service on February 10, 2023, were included in the study. The study population included pediatric (below 18 years), adult (18–50 years), and elderly (over 50 years) patients. The pediatric-aged populations were included by taking into consideration the following health conditions: obesity, mental disorders, and asthma, as well as type 1 diabetes mellitus (DM) among late adolescents due to lifestyle-related factors and the high psychoactive substance use in this age category. Those with medical records with incomplete information on the type of chronic comorbidity diagnosed and the type of cancer were excluded from the study.

### Sample size and sampling procedure

The sample size was calculated using the single population proportion formula under the following statistical assumptions: a 95% confidence level (*Z* = 1.96), a proportion of chronic comorbidities considered to be 0.5, and a 5% degree of precision. Using this formula, the calculated sample size was 384. After adding 10% of the non-response proportion, the final sample size of the study was 422. According to the health information system of the hospital involved in the study, there were a total of 1,567 patients with cancer admitted until February 10, 2023. A simple random sampling technique was employed for the selection of study participants from the total cancer patient records.

### Variables of the study

The outcome variable of the study was the comorbidity status of patients with cancer. A patient who had one of the chronic comorbidities [i.e., hypertension (HTN), DM, CVD, human immune virus (HIV), kidney diseases, anemia, or mental illness] (24) was coded as 1, while those not experiencing any of the aforementioned chronic diseases were coded as 0. The independent variables of the study included age, sex, occupation, marital status, residence, smoking history, alcohol history, *khat* chewing history, body mass index (BMI), cancer type, cancer stage, and chronic disease type. For disease comorbidity, the presence of disease was determined by examining the medical history of the patients. A disease lasting 1 year or more and that requires ongoing medical attention is considered chronic comorbidity. In the current study, the diseases considered as chronic comorbidities were HTN, DM, CVD, HIV, chronic respiratory diseases [e.g., asthma, tuberculosis (TB), chronic obstructive pulmonary disease (COPD), and chronic bronchitis], kidney disease, anemia, and mental illness. Moreover, the use of chemical products was assessed in this study, which included pesticides used as pest control of rodents, insects, or plants and the chemicals in detergents.

### Data extraction procedure and quality control

Data were extracted using a checklist developed through a review of previously published related studies (8, 25–27). Data on cancer patients were extracted from their medical records from their admission to the treatment center on October 1, 2021, up to February 10, 2023. Six health professionals (with a BSc degree) collected the data, while two health professionals (with a master’s degree) supervised the process of data collection. Those in charge of data collection were trained before the process. To ensure the quality of the data, a pretest was conducted on a randomly selected 20% of the participant records. Any error found during the pretest process was corrected, and modifications were made to the final version of the data abstraction format. All collected data were examined for completeness and consistency during data management, storage, and analysis.

### Data processing and statistical analysis

The collected data were entered into a computer using the Epi-Data statistical software version 3.1 and then exported to STATA version 14.2 for further statistical analysis. The categorical variables were described using frequency and percentages, while continuous variables were summarized using the mean with standard deviation. On the top model, the important assumptions of logistic regression, such as chi-square and multicollinearity assumptions, were examined. During variable selection in the building of the model, the following issues were considered: variables of clinical importance, stability of the model, determination of generalizability, and control of confounders (28). Thus, in this study, we purposely included the variables that had *p* < 0.25 in order to include all possible relevant variables with *p* < 0.25, as this cutoff point could include all clinically important variables and confounders. Variables with *p* < 0.25 in the bivariate logistic regression were transferred to multivariate logistic regression. In the multivariable analysis, the strength of the statistical association was calculated using the adjusted odds ratio (AOR) and 95% confidence interval. A *p*-value <0.05 was used to indicate the statistical significance of the factors.

### Ethical consideration

An ethical clearance letter was obtained from Haramaya University, the College of Health and Medical Sciences, and the Institutional Health Research Ethics Review Committee (IHRERC). Official letters of cooperation to conduct the study were sent to HFCSH. Informed, voluntary, written, and signed consent was obtained from the hospital administrators before the data collection. The hospital administrators were also informed that the information obtained from medical records will be kept in complete confidentiality.

## Results

### Socio-demographic characteristics

Of the calculated 422 samples in the study, 419 participant data, with a 99.3% response rate, were included for analysis. The descriptive data analysis illustrated that 64.2% of the patients with cancer were women. Analysis of the age group showed that six out of seven patients with cancer were adults. In addition, 44.15% of the patients with cancer in this study setting had no formal education (**Table 1**).

**Table 1:** Socio-demographic characteristics of cancer patients in Eastern Ethiopia, 2023.

Variables	Frequency	Percent (%)
Sex		
Male	150	35.80
Female	269	64.20
Age category		
Pediatric	17	4.22
Adult	345	85.61
Elders	41	10.17
Religion		
Muslim	282	68.95
Orthodox	107	26.16
Protestant	18	4.40
Others	2	0.49
Current marital status		
Single	52	12.59
Married	329	79.66
Divorced	7	1.69
Widowed	25	6.05
Residence		
Urban	224	53.98
Rural	191	46.02
Educational status		
No formal education	185	44.15
Primary school	112	26.73
Secondary education	81	19.33
College and above	41	9.79
Occupation		
Government employed	44	10.78
Private employed	74	18.14
Farmer	92	22.55
Housewife	137	33.58
Others	61	14.95

### Behavioral and clinical characteristics

Of the total, around 1 of 10 patients with cancer were smokers at baseline. Moreover, at baseline, one-fourth (25.90%) of the study participants were *khat* users. Of the total 419 participants, 15.71% were exposed to any chemical products that are causative agents of chronic disease. Of the 419 diagnosed cancer cases, more than one-fourth (26.49) were breast cancer (**Table 2**).

**Table 2:** Behavioral and clinical characteristics of cancer patients in Eastern Ethiopia, 2023.

Variables	Frequency	Percent (%)
Family history of cancer		
Yes	22	5.91
No	350	94.09
Baseline smoking status		
Yes	44	10.55
No	373	89.45
Baseline drinking status		
Yes	16	3.83
No	402	96.17
Baseline Khat chewing history		
Yes	108	25.90
No	309	74.10
History of using chemical products risk for chronic disease		
Yes	55	15.71
No	295	84.29
History of exposure to X-ray		
Yes	17	4.13
No	395	95.87
Type of cancer		
Breast cancer	111	26.49
Lung cancer	19	4.53
Colorectal cancer	19	4.53
Cervical cancer	38	9.07
Gastric cancer	14	3.34
Hepatocellular cancer	41	9.79
Head and neck cancer	30	7.16
Esophageal cancer	24	5.73
Kaposi sarcoma cancer	4	0.95
Solid tumors cancer	21	5.01
Lymphoma cancer	29	6.92
Endometrial cancer	7	1.67
GTD cancer	14	3.34
Urogenital cancer	10	2.39
Thyroid cancer	2	0.48
Cancer of unknown cause	6	1.43
Ovarian cancer	14	3.34
Anal cancer	3	0.72
Vulvar cancer	2	0.48
Pancreatic cancer	5	1.19
Another type of cancer*	6	1.43
Type treatment provided		
Chemotherapy	265	63.25
Radiotherapy	7	1.67
Surgery	5	1.19
Chemotherapy plus surgery plus hormonal	25	5.97
Chemotherapy plus radiotherapy	30	7.16
Palliative care	17	4.06
Chemotherapy plus palliative	16	3.82
Chemotherapy plus surgery	50	11.93
Others**	4	0.95

*: cancers not mentioned, **: treatment modalities not mentioned.

### Prevalence of chronic comorbidities among cancer patients

**Figure 1:**
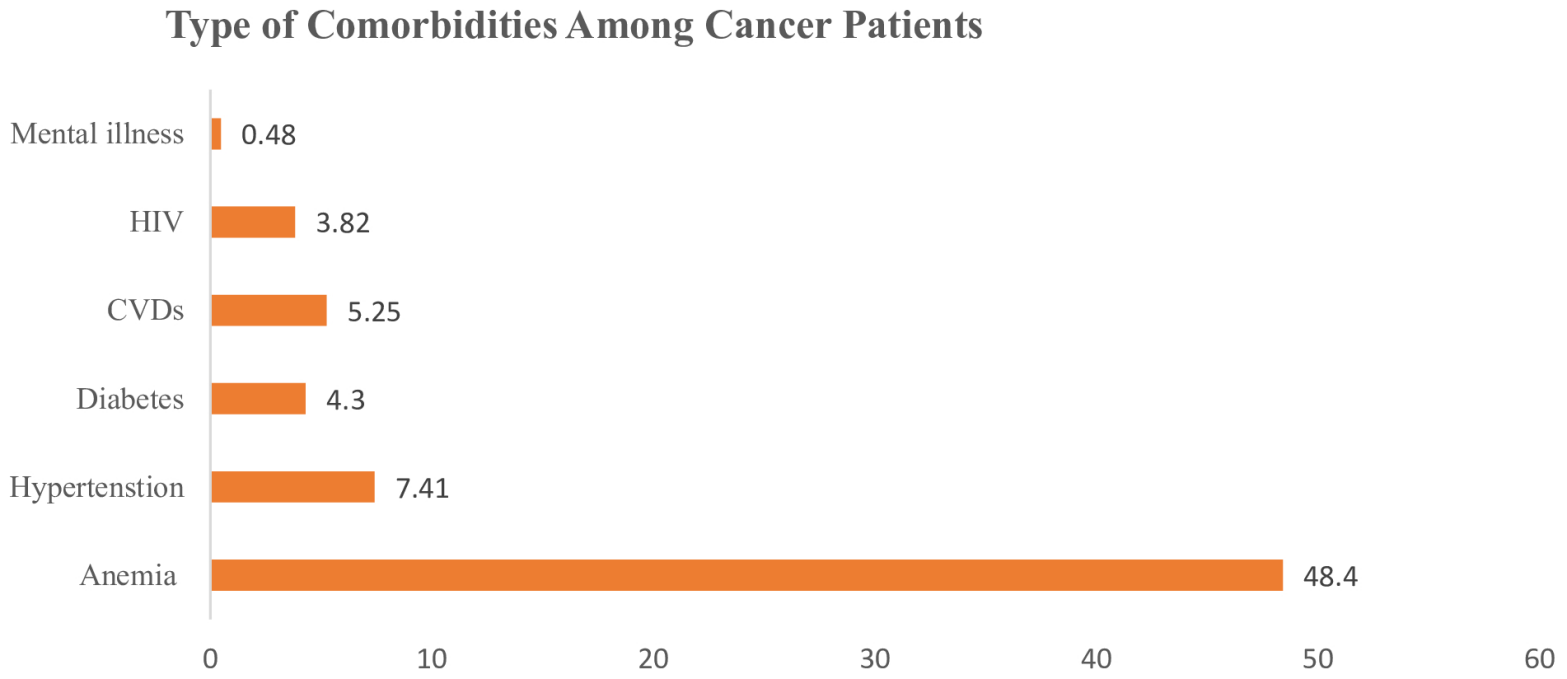
Types of comorbidities and percentages among cancer patients in Eastern Ethiopia, 2023.

Of the total 419 surveyed patients with cancer, 230 (54.8%, 95%CI = 50.0%–59.6%) had at least one comorbidity. Anemia, HTN, and CVD were the most common comorbidities with high prevalence (**Figure 1**).

### Factors associated with comorbidity among cancer patients

A logistic regression model was fitted to identify the factors associated with comorbidities among patients with cancer. In the bivariate logistic regression, sex, residence, age at admission, marital status, education level, occupation, alcohol consumption, and baseline BMI had *p*-values <0.25. In the multivariate logistic regression, the baseline BMI was significantly associated with comorbidities among patients with cancer. The odds of comorbidities were 1.91 (AOR = 1.91, 95%CI = 1.10–3.61) times higher among individuals with obesity when compared with those who had a normal weight (**Table 3**).

**Table 3:** Multi-variable logistic regression of factors associated with comorbidity among cancer patients in Eastern Ethiopia, 2023.

Variables	Comorbidity		AOR with 95% CI	P-Value
	Yes	No		
Sex				
Male	19	131	1	
Female	50	219	1.28 (0.67, 3.47)	0.53
Age category				
Pediatric	2	15	1	
Adult	53	292	0.54 (0.06, 3.76)	0.62
Elders	14	27	1.10 (0.13, 8.24)	0.79
Residence				
Rural	49	175	1	
Urban	20	171	2.37 (0.87, 3.02]	0.06
Marital status				
Single	7	45	1	
Married	50	279	2.33 (0.72, 4.57)	0.46
Divorced and Widowed	12	20	2.59 (0.97, 11.56)	0.16
Educational status				
No formal education	27	158	1	
Primary	18	94	1.25 (0.32, 2.46)	0.32
Secondary	15	66	1.26 (0.41, 2.69)	0.41
College and above	9	32	1.21 (0.47, 3.51)	0.33
Occupation				
Government employed	30	14	1	
Private employed	62	12	0.78 (0.22, 2.27)	0.23
Farmer	84	8	0.66 (0.13, 2.69)	0.15
Housewife	112	25	0.68 (0.20, 2.22)	0.67
Others	51	10	0.90 (0.20, 3.20)	0.71
Body mass index				
Normal	6	97	1	
Underweight	30	154	0.69 (0.17, 2.00)	0.26
Overweight	10	39	1.25 (0.66, 2.86)	0.31
Obese	23	60	1.91 (1.10, 3.61)	0.04*
History of alcohol consumption				
No	6	10	1	
Yes	63	339	2.15 (0.55, 6.14)	0.07

AOR, Adjusted Odds Ratio; CI, Confidence Interval. *: statistically significant factors.

## Discussion

In developing countries such as Ethiopia, there is scarce information on the characteristics of patients with cancer and the coverage of cancer treatment services, including comorbidity screening and prevention strategies. Thus, to shed light on the burden of these comorbidities among patients with cancer, this study aimed to identify the magnitude of and the factors associated with comorbidities in these patients. Accordingly, the present study revealed that, among cancer patients in eastern Ethiopia, the overall prevalence of chronic disease was 54.8% (95%CI = 50.0%–59.6%). In this study, even when it was proposed to include chronic respiratory cases such as asthma, TB, COPD, and chronic bronchitis, none of these cases were identified among the randomly included study participants in the current study. In addition, the study identified that, among cancer cases, a high BMI is associated with the occurrence of chronic comorbidities.

The magnitude of chronic diseases, which was 54.8%, is comparable to the findings from different countries, such as the 47%–62% reported in the Netherlands (29) and the 51.3% reported in the USA (30). However, this result is higher compared to those found in New Zealand (8%–20%) (31) and China (32.8%) (32), but is lower than that reported in Malawi, which ranged up to 90% (8). This discrepancy could be due to differences in the population and the variations in the types of cases considered as comorbidities. For instance, in this study, anemia, HTN, DM, CVD, HIV/AIDS, and psychiatric issues were considered as chronic comorbidities among patients. Although there is a general concept of the occurrence of comorbidities among patients with cancer, there is no clear agreement on the types of cases that should be considered as comorbidities. In addition, the diagnostic approach and resource differences could vary the magnitude of comorbidities among the cases. This explains the disparity in the comorbidity results, with some studies showing a high prevalence and others a lower prevalence (33).

The odds of having chronic comorbidities were higher among cancer patients with obesity compared with patients with normal nutritional status. This finding is in line with those from Malawi (8). It is a fact that obesity is a risk factor for different chronic diseases, including CVD, DM, HTN, and mental illnesses, among others (34–39). Therefore, as obesity is the highest contributing factor to the risk of various chronic diseases, including cancer, patients presenting with the risk of obesity should be monitored closely, as the joint effects of the factors associated with obesity could affect the clinical prognosis of patients with cancer. This study, particularly being one of the very few studies conducted in a poor-resource setup, has limitations. Firstly, the nature of the secondary data used in this study might have prevented the inclusion of all possible variables, resulting in some important variables not being included in the analysis. Particularly, the lack of documentation or resources beneficial for the diagnosis of some comorbidities is a challenge in resource-poor settings, which might have therefore caused underreporting of some chronic comorbidities in this study. Secondly, due to the snapshot nature of the cross-sectional study design, the temporal relationship between the comorbidities and the independent variables could not be assessed. Thirdly, this study is not representative of all patients with cancer in eastern Ethiopia. As a result, many patients might die at home or live with cancer without visiting a health facility due to various factors.

## Conclusion and recommendation

The overall prevalence of comorbidities among patients with cancer was relatively lower than those in previous studies. Obesity was significantly associated with comorbidities. Attention should be given to the burden of chronic comorbidities among patients with cancer through researching, formulating policies, and improving community literacy to manage comorbidities. Thus, interventions for weight reduction and the early detection and treatment of the comorbidities could limit further complications and lower the incidence of other comorbidities.

## Data availability statement

The datasets presented in this study can be found in online repositories. The names of the repository/repositories and accession number(s) can be found in the article/supplementary material.

## Ethics statement

The studies involving humans were approved by Haramaya University College of Health and Medical Sciences Ethical committee. The studies were conducted in accordance with the local legislation and institutional requirements. Written informed consent for participation in this study was provided by the participants’ legal guardians/next of kin. Written informed consent was obtained from the individual(s), and minor(s)’ legal guardian/next of kin, for the publication of any potentially identifiable images or data included in this article.

## Author contributions

AB: Conceptualization, Data curation, Formal analysis, Investigation, Methodology, Project administration, Resources, Software, Supervision, Validation, Visualization, Writing – original draft, Writing – review & editing. MS: Visualization, Writing – review & editing. BH: Visualization, Writing – review & editing. NW: Validation, Visualization, Writing – original draft. MK: Resources, Validation, Visualization, Writing – original draft. SN: Validation, Visualization, Writing – original draft. GA: Data curation, Formal analysis, Methodology, Software, Writing – original draft. YT: Validation, Visualization, Writing – review & editing. SS: Validation, Visualization, Writing – review & editing. BM: Methodology, Supervision, Visualization, Writing – original draft. IM: Supervision, Validation, Visualization, Writing – review & editing.
